# A competency framework for colonoscopy training derived from cognitive task analysis techniques and expert review

**DOI:** 10.1186/s12909-015-0494-z

**Published:** 2015-12-01

**Authors:** Christine M. Zupanc, Robin Burgess-Limerick, Andrew Hill, Stephan Riek, Guy M. Wallis, Annaliese M. Plooy, Mark S. Horswill, Marcus O. Watson, David G. Hewett

**Affiliations:** School of Human Movement Studies, The University of Queensland, Brisbane, Australia; Minerals Industry Safety and Health Centre, The University of Queensland, Brisbane, Australia; School of Psychology, The University of Queensland, Brisbane, Australia; Clinical Skills Development Service, Queensland Health, Brisbane, Australia; School of Medicine, The University of Queensland, Mayne Medical Building, Herston Road, Brisbane, QLD 4006 Australia

**Keywords:** Colonoscopy, Competency, Cognitive tasks analysis, Instructional design

## Abstract

**Background:**

Colonoscopy is a difficult cognitive-perceptual-motor task. Designing an appropriate instructional program for such a task requires an understanding of the knowledge, skills and attitudes underpinning the competency required to perform the task. Cognitive task analysis techniques provide an empirical means of deriving this information.

**Methods:**

Video recording and a think-aloud protocol were conducted while 20 experienced endoscopists performed colonoscopy procedures. “Cued-recall” interviews were also carried out post-procedure with nine of the endoscopists. Analysis of the resulting transcripts employed the constant comparative coding method within a grounded theory framework. The resulting draft competency framework was modified after review during semi-structured interviews conducted with six expert endoscopists.

**Results:**

The proposed colonoscopy competency framework consists of twenty-seven skill, knowledge and attitude components, grouped into six categories (clinical knowledge; colonoscope handling; situation awareness; heuristics and strategies; clinical reasoning; and intra- and inter-personal).

**Conclusions:**

The colonoscopy competency framework provides a principled basis for the design of a training program, and for the design of formative assessment to gauge progress towards attaining the knowledge, skills and attitudes underpinning the achievement of colonoscopy competence.

## Background

Colonoscopy is a difficult cognitive-perceptual-motor task and considerable training is required to achieve competence. The first step in designing an instructional program to guide such training is the identification of the competency or competencies required, and the knowledge, skill and attitude components underpinning each competency [1–4].

The identification of competency components in the medical training literature has typically been achieved through the gathering of expert opinion [1, 2]. For example, in deriving a competency assessment tool for colonoscopy, Sedlack [5] first identified core skills based on review of professional society recommendations and published reviews, and by conducting a focus group of 9 expert endoscopists. The results were then reviewed by a panel that identified 14 skills required to be minimally competent in routine colonoscopy. Another assessment tool for colonoscopy has also been developed on the basis of expert opinion [6], although in this case neither the number of experts involved nor the process followed was reported. More recently, a Delphi panel of 55 experts assisted in the development of a competency assessment tool for colonoscopy, which included 19 checklist items [7]. A related tool for pediatric colonoscopy has also been developed using a Delphi process involving 41 pediatric experts [8].

In each of the above examples, the aim was to develop a method for assessing colonoscopy competence rather than informing instructional design. Accordingly, the results focused primarily on the identification of observable outcomes of competence, rather than the knowledge, skills and attitudes underlying the competency; although the latter two papers included checklist items such as “recognizes loop formation and avoids or reduces appropriately during the procedure (by using pull-back, torque, external pressure, patient position change)” which hint at the underlying perceptual skills, motor skills, heuristics and strategies.

The training committee of the American Society for Gastrointestinal Endoscopy (ASGE) [9] has defined a core curriculum for colonoscopy, which identifies the 25 core motor and cognitive skills required for competence (Table 1). However, no description of the process by which this list of skills was derived was provided.

**Table 1:** American Society for Gastrointestinal Endoscopy core motor and cognitive skills required for competence in colonoscopy [9]

Motor	Cognitive
Correctly holding the colonoscope	Anatomy
Use of the colonoscope controls	Patient selection
Colonoscope insertion	Preparation
Colonoscope advancement	Colonoscope selection
Tip control	Informed consent
Torque	Sedation management
Lumen identification	Assessment of indication and risks
Withdrawal/mucosal inspection	Pathology identification
Loop reduction	Therapeutic device settings
Angulated turns	Integration of findings into management plans
Terminal ileum intubation	Report generation and communication
Biopsy	Complication management
Snare polypectomy	Quality improvement
	Professionalism

### Cognitive tasks analysis

The identification and definition of medical competencies has also been achieved using cognitive task analysis techniques, typically combined with expert opinion methods [1, 2]. Cognitive task analysis involves the systematic study of a workplace to derive descriptions of the cognitive-perceptual-motor processes associated with goal-directed work [10]. A wide variety of data collection methods have been employed, including: observation; “think-aloud” protocols; “cued-recall” interviews involving the use of probe questions; and “critical incident” review interviews. In addition, a range of analytical techniques can be applied to the data depending on the purpose of the analysis. For example, protocol analysis is a qualitative analysis method in which verbalizations, such as responses given during interviews, are classified into functional categories [11].

Cognitive task analysis techniques have commonly been used to identify the knowledge and strategies that characterize expertise in a particular field [12]. They can be used to inform instructional design [13, 14], although in the medical field they have again been more typically used to frame the assessment of competency, rather than to define an instructional framework for use in subsequent curriculum development.

For example, Miskovic et al. [15] conducted semi-structured interviews with seven surgical trainers to identify the skill domains important for the assessment of competency in laparoscopic colorectal surgery. The interviews were recorded and transcribed, and the data categorized. The analysis undertaken was a version of the Delphi method, described by the authors as a “reiterative expert consensus process” (p.477). Categories of responses derived from the interviews were sent to 15 experts who were asked to rate each item with respect to its relevance for summative assessment of competency, and this process was repeated until consistency increased. The final evaluation tool identified 26 characteristics relevant for competent performance.

Yule et al. [16] undertook observations and critical incident review interviews with 27 general, orthopedic and cardiac surgeons to develop a method for assessing surgeons’ non-technical skills. The interviews were recorded and transcribed, before being analyzed using an inductive coding technique derived from grounded theory [17, 18] to develop a skills taxonomy. Characteristics of the grounded theory method include simultaneous data collection and analysis phases, and the creation of codes and categories from the data rather than forcing it into preconceived categories [19]. The analysis resulted in a list of 150 non-technical skills, while the final taxonomy consisted of 14 non-technical skill elements grouped into five superordinate categories.

The teaching of colonoscopy skill has also been the subject of a similar examination. Sullivan et al. [20] video recorded three expert colorectal surgeons while they were engaged in teaching colonoscopy to students at the beginner level. The surgeons were instructed to “think out loud” describing, in particular, the steps and decision points involved in the procedure. This was followed by a period of “free recall” at the conclusion of the procedure. Structured interviews consisting of probe questions derived from reviewing the recordings were subsequently undertaken with each surgeon, and the resulting information was used to construct a 26-step procedural checklist, and 14-point cognitive demands checklist.

Given the importance of a complete understanding of task requirements for optimal design of a colonoscopy training program, the aim of this research was to supplement existing colonoscopy curriculum documents with a competency framework for colonoscopy training based on cognitive task analysis methods combined with expert review.

## Methods

### Study design overview

The investigation was carried out in three metropolitan hospitals (in Sydney, Melbourne and Brisbane, Australia). The cognitive task analysis techniques employed included observation and video recording of live colonoscopy procedures, a think-aloud protocol undertaken during live procedures, and a cued-recall undertaken during post-procedure interviews with a subset of participants. Analysis of the resulting transcripts incorporated aspects of verbal protocol analysis and constant comparative coding within a grounded theory framework. Additional post-analysis interviews were carried out to obtain an expert review of the resulting draft competency framework.

### Participants

Twenty practicing endoscopists (certified by the Australian Conjoint Committee for Recognition of Training in Gastrointestinal Endoscopy) participated in the cognitive task analysis phase. Six expert endoscopists (two of whom also participated in the initial phase) participated in the subsequent expert review process. Ethics approval was granted by the Prince Charles Hospital Human Research Ethics Committee (EC2857) and the University of Queensland Medical Research Ethics Committee (2008001540), and recognized by the participating interstate hospitals. Informed consent was obtained from participating endoscopists, patients and endoscopy assistants.

### Procedures

Two high definition (1080i) video cameras (HDR-FX1; Sony Corporation, Tokyo, Japan) were installed in an endoscopy unit procedure room in each hospital to capture frontal and lateral views of endoscopists performing colonoscopy procedures. High definition digital video recorders (HVR-M25P; Sony Corporation, Tokyo, Japan) were used to record video data from both of the cameras, the luminal view from the endoscopy system (CV-180 Evis Exera II; Olympus Corporation, Tokyo, Japan), and the visualization produced by a colonoscope position detection system (ScopeGuide 3-D Imager; Olympus Corporation, Tokyo, Japan).

Before data collection commenced, each endoscopist was briefed on the “think-aloud” protocol. They were instructed to describe throughout each procedure: their aims/goals and expectations/predictions; their strategies, colonoscope handling techniques and colonoscope shape awareness; cues used for navigation and the detection of abnormalities; and any other thought processes that they are aware of while carrying out the task.

Each endoscopist’s verbalizations were transcribed, and appropriate probe questions identified. A post-procedure interview was carried out with nine of the endoscopists (i.e., all those who indicated that they were available to participate). During these interviews, the endoscopist and the interviewer reviewed audio and video data from the procedure (comprising the luminal view from the colonoscope and the frontal view of the endoscopist) to cue recall. During the session, the endoscopist was encouraged to expand on any information that he/she provided in the “think-aloud”, and answer additional probe questions. The transcript from the interview was then incorporated into the transcript of the procedure.

Analysis of the resulting transcripts employed the constant comparative method within a grounded theory framework [17, 19], commencing with line-by-line coding to identify goals, predictions and strategies for each colonic region before proceeding to allocate the codes to related categories, and eventually develop a draft competency framework. An early draft of the competency framework was based on data from the first ten participants [21]; however, it was revised substantially once all of the data from the cognitive task analysis phase had been analyzed. Semi-structured interviews were then carried out with six expert endoscopists and further adjustments to the draft competency framework were made as a consequence of the feedback received.

Modifications arising from this review process included the addition of two components to the situation awareness category (i.e., awareness “of anatomical constraints” and “for correct equipment functioning”). In addition, several components in the “Clinical reasoning” category were reorganized. Specifically, three of the components (i.e., “procedural decisions”, “therapeutic decisions” and “problem solving”) were combined into two (i.e., “procedural decisions/problem solving” and “therapeutic decisions/problem solving”). Apart from these final changes, the draft subjected to expert review was identical to the proposed framework described below.

## Results

**Fig. 1:**
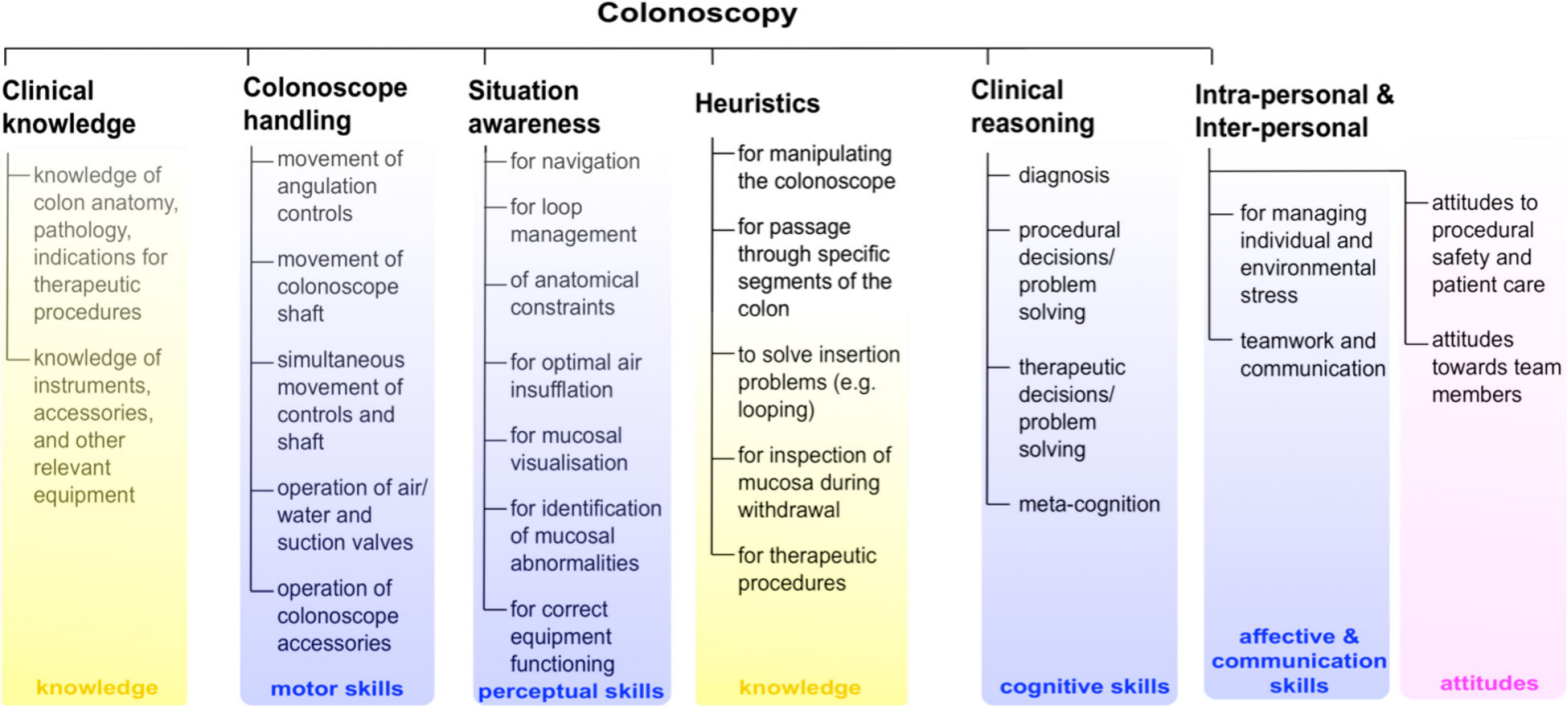
Proposed colonoscopy competency framework derived from cognitive task analysis techniques and expert review

Twenty-seven competency components (knowledge, skills and attitudes) were identified and grouped into six categories (clinical knowledge; colonoscope handling; situation awareness; heuristics and strategies; clinical reasoning; and intra- and inter-personal) within the Colonoscopy Competency Framework (Fig. 1).

### Clinical knowledge

Colonoscopy-specific clinical knowledge refers to explicit knowledge about colorectal anatomy, pathology, treatment, and patient medical history characteristics, and the implications of this knowledge for the colonoscopy procedure. Examples from the transcripts illustrating such knowledge include the following:

“If there’s chronic constipation, and there’s a history of laxative use then that often suggests that they’re going to have a redundant colon that will be difficult to get around.”

“He’s got quite significant rectal inflammation which is a result of colitis, so that immediately, this is a red flag to me about the way I’m going to conduct this procedure because of the potential risk of inflammation throughout the rest of the colon.

“I’m going to start off with a rectal examination first looking for any perianal disease… You can exclude any anal pathology that might preclude colonoscopy, such as from Crohn’s disease or any other surgery that the patient might have had”.

Although clinical knowledge is required for colonoscopic insertion, it was particularly evident during withdrawal as a resource to draw upon during diagnosis and treatment of any pathologies detected. For example:

“Loss of vascular pattern is a helpful sign… I think you can see a slight redness to the mucosa and that’s what I look for…unless it is a sessile adenoma or a hyperplastic polyp. They tend to look a bit paler because they’ve got mucus on them, which makes them paler.”

“That’s an angioectasia in her ascending colon and if she were anaemic or anything I’d treat that, but it’s altered bowel habit that we’re doing this for… Just wonder if we should do it… She’s going back on aspirin and Plavix; let’s treat that…”

“If it starts to bleed, it’s going to bleed big time. Again, I’m just going to use the snare to look at it, and we’ve got adrenalin in the room, not that it’s going to be much help in this setting because if that sucker wants to bleed it’s going to bleed…”

Endoscopists also displayed knowledge about various items of colonoscopy-related equipment, including colonoscopes and their accessories. This knowledge includes the advantages and disadvantages of particular items, and how and when to use them appropriately. For example:

“I prefer to use a pediatric scope with severe diverticular disease. You can get quite significant colonic stenosis and narrowing; it’s much easier to get through…”

It was also noted that a sufficient technical understanding was required to enable the endoscopist to trouble-shoot in the case of equipment failure, and to know when repairs were required.

### Colonoscope handling

The colonoscope handling category refers to the motor skills required to coordinate precise manipulations of the controls located on the colonoscope (i.e., the up/down and right/left angulation control wheels, and the air, water and suction valves, which are all typically manipulated with the left hand), with controlled movements of the colonoscope shaft (i.e., forwards, backwards, and torque movements) simultaneously executed via the right hand. To achieve a task-related goal, the endoscopist often uses their left hand to precisely control movement of the colonoscope tip via the angulation controls, while simultaneously using their right hand to apply forces in a variety of directions to the colonoscope shaft. For example:

“I use a combination of quite aggressive angulation control on my left hand, both up/down and left/right, and my right hand puts torque on the instrument… and I’m using left hand to maintain a view.”

“It’s a combination of both anti-clockwise torque and delicate up/down control with the left hand to try to get the scope to go where I want it to.”

“Using both torque and the controls in my left hand to try to steer myself through this area of severe diverticular disease, using very little pressure in terms of pushing forward. I’ve got the scope between the top of my thumb and third finger, just easing it forward very gently.”

“So, on withdrawal, it’s mainly just your large wheel – your up/down – that you’re moving up and down around the various bends and folds; it’s just gentle torqueing left and right… With air aspiration, it’s a gradual thing. You just tap on the air button; you don’t leave it on.”

Experienced endoscopists displayed a high degree of automaticity of these skills.

### Situation awareness

Situation awareness is the ability to integrate relevant sensory cues into an accurate perception of the current and likely future states of the dynamic environment in which action takes place [22]. Situation awareness is a necessary but not sufficient condition for correct skill execution. One way in which experienced endoscopists display situation awareness is in their perception and understanding of the colonoscope tip’s location within the colonic tract. This understanding, which relies primarily on the use of visual cues, is required during insertion to guide movement of the colonoscope and its tip, and to plan for the next stage of insertion. Colonoscopists also utilize their knowledge of the anatomical constraints and physical characteristics of the various components of the colon to help interpret the perceptual information. For example, visual cues may indicate the occurrence of colonic spasms, the presence of a redundant colon, the thinness of the colonic mucosa, or the presence of a sharply angulated region of the colon ahead. Examples from the transcripts included the following:

“The landmarks for identifying the lumen are generally darker spots. You use the luminal folds, which you can see here are actually transverse circular folds, and in that case the lumen is always perpendicular to those.”

“In the sigmoid, you have to be quite clear that you are going for the lumen not into a diverticular pocket, which can be quite misleading.”

“There’s pooling of fluid, which gives you a clue that you’ve been in the descending colon…and secondly you get a changing of the nature of the folds. You see that they start to get triangular, and you’ve turned the corner; this is very typical of the splenic flexure.”

“I know I’m in the caecum because I can see the ileocaecal valve.”

“We’re actually at the hepatic flexure here because you can see the transmural blue tinge of the liver.”

Endoscopists must also be aware of the amount of tension exerted on the colon by the colonoscope because of its shape; that is, whether the colonoscope is straight/neutral, or whether there is some degree of looping. Both visual and haptic cues are used to obtain this information. For example:

“I start to notice the scope is looping. I know that because of a loss of one-to-one relationship between the scope and the image on the screen.”

“You can see on the ScopeGuide that I’ve got a looped scope here… I’m going to have to carry this loop here anyway, so I’m going to continue to push in and be aware that I’m forming a loop in the sigmoid colon.”

“Often, as you’re rotating the scope, you’re torqueing and withdrawing, you feel, it’s almost like it clunks. Suddenly, all the tension goes and it goes clunk… You feel it almost snapping into being straight… That’s quite useful because you just know that you’re straight.”

Safe practice also requires that there is an appropriate amount of air in the colon during both insertion and withdrawal. Endoscopists maintain an awareness of how much air has been inserted into the colon, and carry out actions to correct the degree of insufflation if an appropriate balance is not maintained. Although visual cues are important, they may not be sufficient because air moves within the colon and can collect in other regions not currently being visualized. In the withdrawal phase, it was noted that it is also important to maintain an appropriate degree of insufflation for adequate and complete mucosal inspection.

During withdrawal, endoscopists also maintain an awareness of which areas of the colonic wall have been visualized to ensure that all areas of the mucosa are inspected thoroughly. Endoscopists rely on visual cues (such as the rate at which the colon wall appears to “move past” the tip camera) to monitor the speed and smoothness of their withdrawal. In addition, anatomical cues are used as landmarks to allow the endoscopist to maintain awareness of which areas have been visualized.

Whilst particular inspection techniques can optimize mucosal visualization during withdrawal, the endoscopist also needs to recognize when more detailed viewing is required. Endoscopists use explicit knowledge of visual cues relating to the shape, color and textural patterns of visualized features to prompt closer examination for diagnosis. For example:

“What we’ve all developed during our training is an understanding of what a normal mucosa looks like…and that’s pattern recognition.... If I get even closer, you can actually see the little dots… We are looking for permutations or perturbation of those mucosal appearances.... You also look for changes in the light reflex and for changes in the colour.”

“Sessile serrated polyps can be difficult to find because they’re often quite flat, and the secret is seeing the mucus they secrete.”

“You can see here a nice example of arteriole and venule… You see how they tend to coexist like that; you’re looking for distortion of those patterns.”

### Heuristics and strategies

Here, the term “heuristics” is used to refer to explicit knowledge in the form of “rules of thumb” or “tricks of the trade” [23], which sometimes but not always achieve the desired outcome; while strategies are “rule-based methods” [24] which are routinely employed to achieve a given sub-task. The endoscopists described a wide range of heuristics and strategies learned from teaching texts, instructors, or personal ‘trial and error’. Some of these approaches could be used during simple routine colonoscopy procedures, while others were specific to difficult situations encountered during insertion or therapy.

Some strategies involved applying a simple technique to achieve a specific goal. For example, one participant described a technique for getting past folds in the following terms: “Just massage our way through that and, as soon as I can see lumen, I suck.” Another described a technique of repeatedly advancing and then pulling back and straightening to concertina the colon onto the scope. Participants also described heuristics that involved changing the patient’s position and applying abdominal pressure in different locations to facilitate insertion at different stages of the procedure. Other strategies for insertion, as illustrated in the following examples, were more complex or situation specific:

“Your basic straightening manoeuvre is pulling back with clockwise rotation. It appears to work quite well in the transverse colon… The other thing to remember is that not all loops will be relieved by clockwise torque. So try counter-clockwise and if you’re getting minimal resistance, the scope’s staying in a stable position, then that sometimes helps you.”

“We’re up against the flexure here. What I often try and do here is back away to get a good view of what’s going on, take out a bit of air, come up, follow this flexure around… Sometimes you can back into it, so max tip down… We’re at a point here where we need to make a decision of whether to use pressure or not. Usually, I’ll give it one go of going a little bit long and if it slides, that’s all right… If not, we need to get some pressure on.”

“It was too uncomfortable for him, and I wasn’t going anywhere pushing in because of the loop. So I tried something else, which is withdrawing whilst trying not to lose any ground. So I’m just trying withdrawing and clockwise twist whilst trying to remain in the lumen.”

“A very useful technique is to roll the patient on their right side because it opens up the splenic flexure it makes it much easier to get through…Then, once you’re around it and into the transverse, you can roll them back on their left side.”

“I haven’t got one-to-one movement, so I’m going to use some clockwise torque and pull back again to try and regain as straight a scope as possible to remove any loops before I try and proceed again… If that doesn’t work a second time, then I’ll try some sigmoid pressure.”

“You’re going to use suction and rotation to get as far around as you can, but once you reach a point here where you can’t pull back any more, then some cautious inwards pressure, accompanied by a bit of rotation… Then, if that doesn’t work, I get some pressure on.”

“There’s two approaches to doing that. One is to carefully try to bring it around so you maintain a view at all times… The other approach is just to bring it around like I did then, get a stable scope position, and then reassess.”

“If you advance the scope and you’re not going anywhere, you need to change pressure right away.”

“I’ve just had to reorient the scope and pull back with right twist to allow that typical anterior spiral or alpha loop to reduce before I can go further forward.”

“At this point, there are a few different options for moving forward… Initially, what I usually do is just turn the corner and pull back somewhat, and suck to make sure that I’ve got a straight scope and a short scope… At this point, I try to relax the up/down control slightly so you don’t form what some call a hockey stick at the splenic flexure, and then push the scope gently forward.”

“If I try to shorten the scope, I can keep some right torque on this point which keeps the lumen… The general strategy that I use is to keep the lumen on my right… which allows me to try and maintain a straight scope as we traverse through the sigmoid colon....because, as you go through the sigmoid colon, the instrument wants to loop.... I’m having trouble finding the lumen. It’s twisted around. I may not be able to keep it on my right. It’s actually straight ahead of us up to the right, so there we go. I’ve just pulled back and I’ve sucked, and now I’m torqueing to the right.”

“I’m going to have to reduce this loop because I can’t seem to get it to go further forward. I’m going to try and reduce this here by a combination of suction and torqueing.”

A range of strategies were also described for withdrawal and therapy. For example:

“Now I pull back, and I undertake a circular rotatory inspection technique looking behind each of these folds; and in doing so I often need to flatten out each of those folds and make sure I look behind…”

“I’ll show you my way of doing it… Open up halfway now ‘till the snare opens up laterally. Keeping going now. It opens up wide there. Stop there. I’m just going to lay the snare down on the polyp. Just a matter of maintaining good luminal views with the position of the scope. Now, I want you to close and cut through now.”

### Clinical reasoning

Clinical reasoning is the set of cognitive processes that underlie clinical diagnosis and management [25–27]. Three main “problem spaces” can be identified for the colonoscopy task: insertion of the colonoscope to caecum; mucosal inspection during withdrawal; and therapeutic procedures. Working within each of these problem spaces requires a different mix of knowledge and skills, and consequently, effective reasoning may rely on different types of thinking skills. For example, during withdrawal, decisions were often made about when and how to inspect particular regions of the mucosa that may be especially difficult to visualize. Diagnosis involved using knowledge about abnormal pathology and the available visual cues to make clinical judgments. During therapy, numerous decisions were made, for example, relating to the appropriate choice of intervention or colonoscope accessory, and the optimal position of the colonoscope relative to the anomaly for use of the chosen accessory. The following are examples of clinical reasoning from the transcripts:

“There’s a small little polyp there, which we’ll get on withdrawal. In fact, no we won’t; we’ll take that off now… The reason I’m doing that now is that I’m not entirely sure where we are and in the distal sigmoid it can be difficult to find it again.”

“Another thing that can be a problem in the area, particularly when there’s severe diverticular disease, is some bowel spasm, and sometimes in this situation I’ll use Buscopan.”

“What I’m looking for here is the typical extrusion of fat that falls out of the lipoma when you biopsy them repeatedly.”

“Generally, with big polyps, we take them off on the way back… Diathermy can make the wall of the colon weak, and if you are pushing and you get a loop where that polyp site is you can cause more damage.”

“You can get polyps in the diverticulae. They are really tricky because the walls in the pockets tend to be very thin walls. You have to be so careful that you don’t perforate in that area.”

### Intra-personal and inter-personal

Intra-personal and inter-personal competency components refer to the affective and communication skills, and attitudes, required for safe and efficient colonoscopy*.* Current models of work stress emphasize the interaction of environmental and individual factors, and suggest that (as part of a holistic approach to the management of work stressors) the individual needs to develop coping skills to manage maladaptive emotional reactions to task stressors [28]. The analysis identified the need for endoscopists to manage short-term emotional and behavioral responses (e.g., irritation or frustration) to stress factors intrinsic to colonoscopy. These include difficult colonoscopy procedures, task uncertainty, time pressure, and carrying the burden of responsibility for patient safety and decision making.

Competent endoscopists reduce the negative impact of task stressors on their performance by using strategies that can be classified as either behavioral (e.g., “stepping away” from the task for a moment) or cognitive (e.g., mentally reminding oneself of the primary goal of the task). For example, one participant explained:

“I do remember getting frustrated a lot when I wasn’t as good technically… Even these days, on very hard ones, I’ll throw the scope down and swear, but I get that out and I just get on and get it done. One of the things I’ve learned is that you’ve got to take each colon as it comes; you can’t be thinking ‘oh I’ve got to get the colon done in 20 minutes because the next one’s going to be waiting for me.’ The colon is going to take as long as it’s going to take, and you’ve just got to accept that and go with it.

Although teamwork and communication skills are required in all interactions between the endoscopist, nurse/assistants, and other allied professions participating in the task, these skills become more crucial during difficult therapy. Not only does good teamwork and communication facilitate effective equipment handling (e.g., in using colonoscope accessories, and other associated equipment), but excellent team skills can result in better decision making during complex procedures [29]. Examples from the transcripts which illustrate these issues include the following:

“This is where it is vital that the nursing staff understand the equipment and can act if there is a problem straight away. You’re approaching a point of no return, that this polyp has got to come off.”

“I need a bit of pressure here. [To nurse:] Can I have some low pressure on the left side? I want you to lift up… Just take your pressure off for a minute… Let’s get some slightly higher on the left side, pressure now.... Just relax… Can I have some left sided pressure, higher up on the left side this time?… Okay, that’s good. Relax.”

“The idea is to get the scope straight and then put pressure where the loop forms to stop that from happening. Some nurses are very good at it… Some of the nurses don’t really understand what they are doing.”

Appropriate attitudes towards procedural safety and patient care are one of the most important competency components for colonoscopy; it was apparent during analysis of the transcripts that these attitudes underpin most, if not all, behaviors and reasoning processes undertaken during the procedure. Concern for patient safety and discomfort were expressed by the participants during difficult insertions, and during withdrawals when decisions were made with regard to abnormalities that had been detected and the selection of an appropriate treatment. For example:

“One of the challenges is balancing the amount of air that you’ve put in to the colon… Unfortunately, with some patients, you do end up having to put too much air in, which means it’s quite uncomfortable for them.”

“At the very end of the procedure, I take all the air out and it makes the patient more comfortable.”

“You try, if at all possible, when you’re taking the polyp off (to) minimize the chance of any collateral burns… It’s all about minimizing risk.”

“It’s all about being very gentle… You want to minimize the amount of trauma or force you’re putting on the bowel, so if you’re having to torque too hard you need to be looking at what do you do differently. Do I need to roll them? Do I need to put pressure on?…”

“You have to respect the caecum. It’s only very thin… The last thing you want to do is perforate.”

## Discussion

In this study, a range of cognitive task analysis methods (specifically: observation, a think-aloud protocol and cued-recall) were employed to identify the competency components exhibited by practicing endoscopists, and the findings were subsequently refined through an expert review process. Ultimately, a proposed colonoscopy competency framework was identified comprising twenty-seven competency components grouped into six categories: clinical knowledge; colonoscope handling; situation awareness; heuristics and strategies; clinical reasoning; and intra-personal and inter-personal (Fig. 1). Our primary aim was to provide a principled basis for future instructional design with respect to colonoscopy training.

### Comparison with the ASGE Core Curriculum for Colonoscopy and the ASGE Assessment of Competency in Endoscopy, Colonoscopy Skills Assessment Tool

The ASGE Core Curriculum for Colonoscopy [9] identifies 25 core motor and cognitive skills required to be competent in colonoscopy (Table 1). As might be anticipated, there is a considerable degree of overlap between the ASGE list of core skills and the knowledge, skills and attitudes identified within the proposed colonoscopy competency framework derived during the current research. In particular, both documents emphasize the fundamental importance of colonoscope handling skills; anatomical knowledge; and clinical reasoning for diagnosis and therapy.

The ASGE Assessment of Competency in Endoscopy, Colonoscopy Skills Assessment Tool [30] is a version of the Mayo Colonoscopy Skills Assessment Tool [5] modified by the ASGE Training Committee. The assessment tool includes 11 topics (Knowledge of patient medical history; Management of patient discomfort; Use of air, water and suction; Lumen identification; Scope steering during insertion; Fine tip control; Loop reduction techniques; Furthest landmark reached; Mucosa visualisation during withdrawal; Pathology identification; Polyp detection; Location of lesion; Interventions performed) and two overall assessment questions addressing motor skills and cognitive skills (including situation awareness). Again, there is a considerable degree of congruency between the topics included in the Colonoscopy Skills Assessment Tool and the components of colonoscopy competence identified here.

However, the colonoscopy competency framework proposed here more explicitly identifies the importance of perceptual skills and the integration of the information so gained into accurate situation awareness throughout the procedure. While the ASGE core curriculum describes a number of heuristics and strategies, such as the use of external transabdominal pressure and changes in patient position to assist advancement of the colonoscope, the framework described here places greater emphasis on the importance of providing training in the full range of heuristics and strategies for insertion, inspection and therapeutic procedures. Similarly, while the AGSE curriculum document notes the importance of teamwork and communication with an assistant, the current research explicitly identifies these aspects within the broader category of intra-personal and inter-personal skills and attitudes. These aspects of competence are not addressed within the Colonoscopy Skills Assessment Tool.

### Potential applications of the colonoscopy competency framework

In general terms, the proposed colonoscopy competency framework provides a principled basis for the design of training programs and formative assessments; however, there are numerous ways that these ends may be achieved in practice. For instance, at the most minimal level, the framework could simply be used to inform potential revisions to existing curricula and competency assessment tools by identifying any gaps that remain to be filled.

A more ambitious approach would be to develop a comprehensive set of highly-focused training modules and assessments, each targeting a specific competency component. This may be of particular benefit for components for which expertise accumulates relatively slowly in clinical settings, such as the identification of mucosal abnormalities. Indeed, a prospective multi-center study found that the polyp detection rate of gastroenterology fellows did not increase over the course of their first 150 cases (despite improvements in other outcome measures, such as time to caecum and the caecal intubation rate) [31]. Therefore, a plausible alternative approach in relation to this competency component may be to develop a novel computer-based training methodology that, in a relatively short space of time, gives the trainee the equivalent of a career’s worth of exposure to and practice at identifying abnormalities, in conjunction with timely and accurate performance feedback. However, such an approach would require a significant investment in the development and validation of the training materials and assessments.

Because the proposed framework focuses on competencies rather than outcomes, it does not prescribe the required form of assessment or feedback. One advantage of this approach is that it permits training and evaluation to be conducted by methods other than live colonoscopy, potentially reducing risks to patient safety (especially during the earlier phases of skill acquisition). Simulation-based training, for example, has the potential to accelerate skill acquisition if it addresses one or more of the competency components in the proposed framework. At present, no commercially-available colonoscopy simulation device addresses all of the competency components, heuristics and strategies that we have identified [32]; hence, a potential application of our findings would be the development of more advanced virtual reality colonoscopy simulators with more comprehensive curricula to better aid in the teaching of colonoscopy. An advantage of such devices is that they have the potential to provide feedback on metrics that can be measured via the technology but not observed by a human assessor. However, colonoscopy simulators and other forms of simulation-based training require validation evidence to establish that the skills acquired transfer to real procedures. Our prediction is that trainees who receive effective training in all components of the proposed framework will achieve more rapid skill acquisition than those trained by traditional methods, even when evaluated using existing measures that focus on outcomes (such as the ASGE assessment tool).

### Limitations

The scope of the investigation is restricted to the colonoscopy procedure itself and does not consider pre-procedural components (e.g., obtaining informed consent) or post-procedural components (e.g., discussion of findings and documentation). In addition, it would arguably have been preferable if all six of the experts who participated in the expert review process had been independent of the sample who participated in the cognitive task analysis phase (rather than four).

## Conclusion

Cognitive task analysis methods (observation, a think-aloud protocol and cued-recall) and subsequent expert review were employed to identify the competency components exhibited by practicing endoscopists with the aim of providing a basis for future instructional design. A colonoscopy competency framework was identified consisting of twenty-seven competency components grouped into six categories: clinical knowledge; colonoscope handling; situation awareness; heuristics and strategies; clinical reasoning; and intra- and inter-personal. The framework provides a principled basis for the design of a colonoscopy training program, and for the design of formative assessment to gauge progress towards attaining the knowledge, skills and attitudes underpinning the achievement of colonoscopy competence.
